# Development and Characterisation of a Human Chronic Skin Wound Cell Line—Towards an Alternative for Animal Experimentation

**DOI:** 10.3390/ijms19041001

**Published:** 2018-03-27

**Authors:** Matthew Caley, Ivan B. Wall, Matthew Peake, David Kipling, Peter Giles, David W. Thomas, Phil Stephens

**Affiliations:** 1 Stem Cells, Wound Repair & Regeneration Group, Cardiff Institute of Tissue Engineering and Repair, Oral and Biomedical Sciences, School of Dentistry, Cardiff University, Cardiff CF14 4XY, UK; m.caley@qmul.ac.uk (M.C.); i.wall@aston.ac.uk (I.B.W.); matthew.peake@newcastle.ac.uk (M.P.); 2 Division of Cancer and Genetics, School of Medicine, Cardiff University, Cardiff CF14 4XN, UK; KiplingD@cardiff.ac.uk; 3 Division of Medical Genetics, School of Medicine, Cardiff University, Cardiff CF14 4XN, UK; gilespj@cardiff.ac.uk; 4 Advanced Therapies Group, Cardiff Institute of Tissue Engineering and Repair, Oral and Biomedical Sciences, School of Dentistry, Cardiff University, Cardiff CF14 4XY, UK; ThomasDW2@cardiff.ac.uk

**Keywords:** wound healing, fibroblasts, chronic wound, microarray, animal alternative

## Abstract

**Background**: Chronic skin wounds are a growing financial burden for healthcare providers, causing discomfort/immobility to patients. Whilst animal chronic wound models have been developed to allow for mechanistic studies and to develop/test potential therapies, such systems are not good representations of the human chronic wound state. As an alternative, human chronic wound fibroblasts (CWFs) have permitted an insight into the dysfunctional cellular mechanisms that are associated with these wounds. However, such cells strains have a limited replicative lifespan and therefore a limited reproducibility/usefulness. **Objectives**: To develop/characterise immortalised cell lines of CWF and patient-matched normal fibroblasts (NFs). **Methods and Results**: Immortalisation with human telomerase resulted in both CWF and NF proliferating well beyond their replicative senescence end-point (respective cell strains senesced as normal). Gene expression analysis demonstrated that, whilst proliferation-associated genes were up-regulated in the cell lines (as would be expected), the immortalisation process did not significantly affect the disease-specific genotype. Immortalised CWF (as compared to NF) also retained a distinct impairment in their wound repopulation potential (in line with CWF cell strains). **Conclusions**: These novel CWF cell lines are a credible animal alternative and could be a valuable research tool for understanding both the aetiology of chronic skin wounds and for therapeutic pre-screening.

## 1. Introduction

Chronic skin wounds are an ever-increasing burden to health care providers. In the United States (US) alone between 2.4 and 4.5 million individuals suffer from chronic wounds with associated treatment costs of $10 s billions [1,2,3]. Within the United Kingdom (UK), the National Health Service treats hundreds of thousands of patients with chronic non-healing wounds, which amounts to an annual cost of between £2.3 bn and £3.1 bn [4,5,6,7,8]. As well as being expensive to treat and manage, chronic wounds significantly reduce the quality of life of those who suffer with them. Unfortunately, the prevalence of chronic wounds increases with age, suggesting a significant looming health and financial problem as our society ages.

In an attempt to study chronic wound biology and to test for potentially efficacious treatments the scientific community has turned to the utilisation of chronic wound animal model systems. However, to generate such animal model systems the conditions that are associated with formation of the chronic wound are often artificial and ineffective. For example, to model the venous leg ulcer microenvironment local tissue ischemia is generated often on the ears or back skin of animals. Ischemic back wounds in rats can be generated using H shaped incisions, reducing blood perfusion along the horizontal wound [9,10,11]. Modifications to the basic skin flap ischemic wound model have also been reported, such as the insertion of a silicone sheet under the skin flap to reduce blood perfusion [12,13,14,15,16,17,18,19]. Ischemic ear models can also be generated by surgical division of the arteries of the ear [16,20,21]. However, there is a significant problem with these model systems in that whilst they do represent an impaired healing system, they do not create wounds that fail to heal and so do not truly replicate the human chronic venous ulcer situation. Thus, the existing animal model systems provide useful tools for studying impaired wound healing, but more accurately model delayed acute wound healing rather than true chronic wounds.

In order to provide details about the biological mechanisms that are associated with the study of chronic wounds, chronic wound fibroblasts (CWF) have been isolated and utilised to gain an insight into the cellular mechanisms as to why such wounds fail to heal. CWF have a distinct phenotype that is retained during in vitro culture which relates to the failure of chronic wounds to heal. For example, when compared to fibroblasts taken from acute wounds or normal skin (NF), patient-matched CWF have a reduced proliferative potential [22,23,24,25,26], an altered morphology (appearing polygonal with clear actin stress fibres and they are also larger than NF [27]) and a reduced ability to migrate and repopulate an experimental wound [22,26,28,29]. As well as phenotypic alterations, gene expression profiles are also altered in CWF when compared to NF [30,31,32,33]. For example, fibronectin gene expression was demonstrated to be up-regulated in CWF as compared to patient-matched NF when cultured in vitro [34], which correlates with in vivo observations [35].

As with all primary cells [36,37,38], CWF isolated directly from patients have a finite lifespan in culture [22,24] and will change their cellular responses as they become more aged thereby, limiting their utility as a reproducible model for studying chronic wound biology. However, for a number of cell types, immortalised lines (often through the over expression of human telomerase reverse transcriptase; hTERT) are now available that retain their specific genotype/phenotype [39,40,41,42,43]. Whilst there are some reported examples of immortalised skin cells [44,45], to date there are no reported, validated human chronic skin wound/disease cell lines. Hence, the aim of this study, was to characterise patient-matched CWF (as compared to NF) hTERT-immortalised cell lines to investigate whether they retained their disease-specific phenotype and could potentially, in the future, be utilised as a model cell line for therapeutic and cytotoxicity testing of candidate drugs for the treatment of chronic wounds, thereby reducing unnecessary animal experimentation.

## 2. Results

### 2.1. Introduction of hTERT Allows Primary NF and CWF Cell Strains to Escape Replicative Senescence

**Figure 1:**
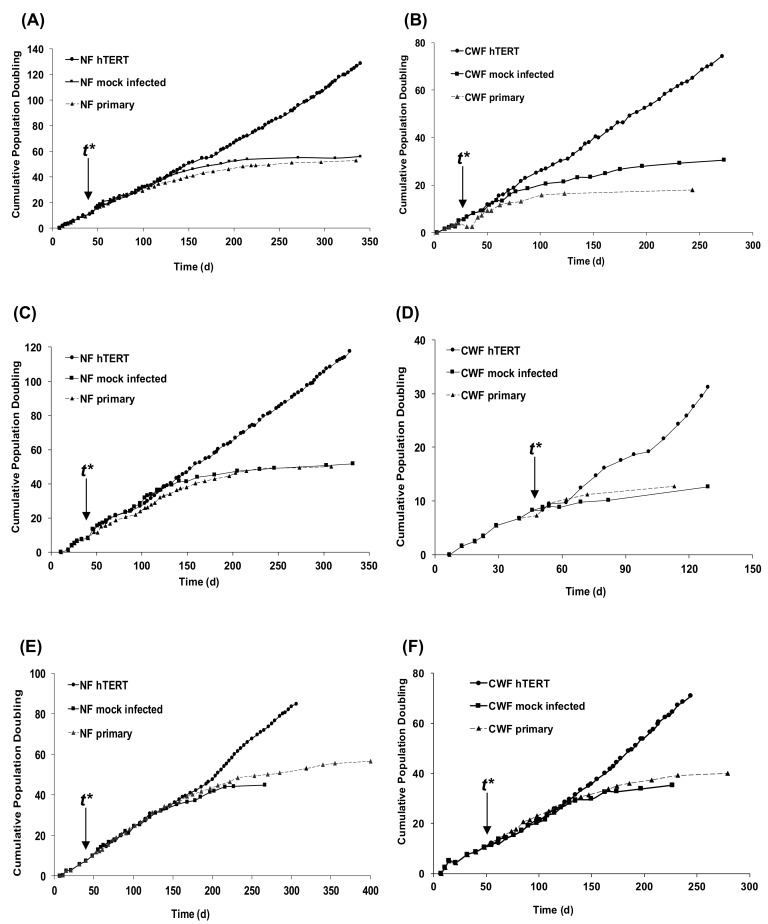
Cumulative population doublings for normal skin (NF) (**A**,**C**,**E**) and chronic wound fibroblasts (CWF) (**B**,**D**,**F**) primary cells (▲), mock-infected cells (■) and catalytic component (hTERT)-infected cells (●) from Patient 1 (**A**,**B**), Patient 2 (**C**,**D**) and Patient 3 (**E**,**F**).

Patient-matched fibroblast strains were isolated from the chronic venous leg ulcer wound bed and healthy, uninjured dermis of three patients as reported previously [22]. Cells were cultured for extended periods of time until they underwent replicative senescence (as evidenced by the plateauing of the growth curve). For all of the patients examined, CWF primary cell strains consistently underwent replicative senescence after a shorter duration of time in culture and at lower PD than their corresponding, patient-matched NF primary cell strains (Figure 1).

hTERT-immortalized cell lines were established for both CWF and NF by infecting cells at time t* (indicated in Figure 1) with a retrovirus carrying the gene encoding hTERT along with the puromycin resistance gene for the selection of the cells ectopically expressing active telomerase. To control for the infection procedure, “mock infected” cells were prepared by infecting cells with an empty vector (containing the puromycin resistance gene) only. For all of the patients examined a notable extension of lifespan was achieved for all populations of CWF-hTERT and NF-hTERT. None of the cells lines demonstrated any signs of a slowing of their growth rate, a response that would have been indicative of them entering senescence. Mock-infected cells all underwent replicative senescence at similar times to their non-infected primary cell strains. Despite demonstrating linear growth in all three patients, overall the hTERT immortalised CWF demonstrated reduced population doubling rates when compared to their patient matched hTERT-NF; a similar differential growth rate observed for the respective non-immortalised cell strains and the control-infected cells (Table 1).

### 2.2. Immortalised Cell Lines Express hTERT and hTR and Have Active Telomerase

**Figure 2:**
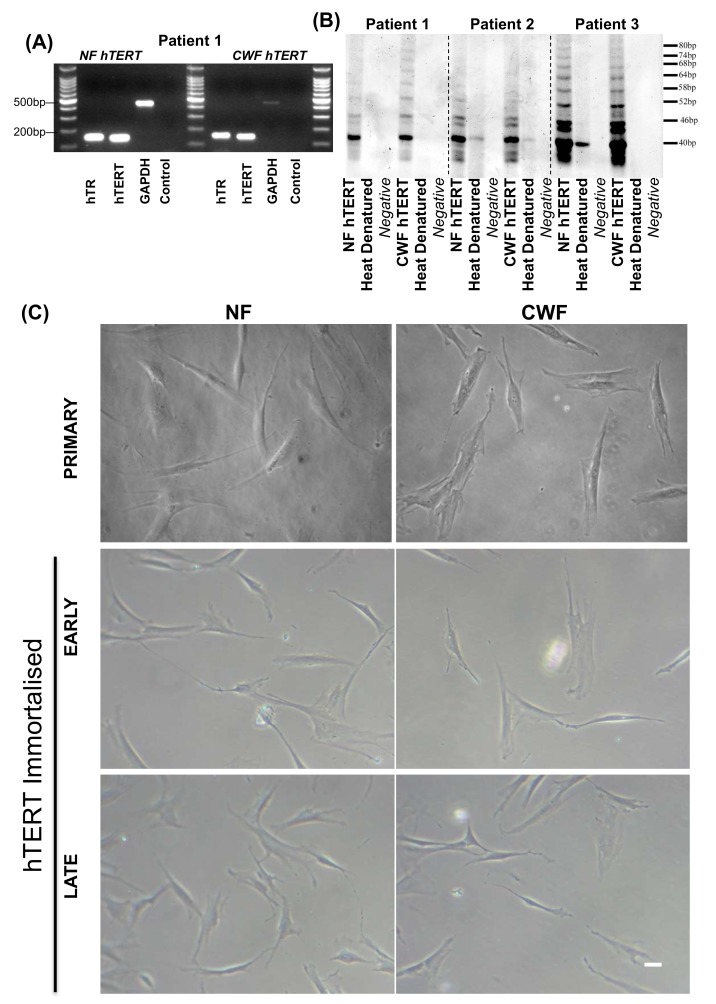
Immortalised NF and CWF cell lines express hTERT and template component (hTR) as demonstrated by RT-PCR (**A**) and have active telomerase as demonstrated by the appearance of a 6 bp ladder (**B**). CWF immortalisation and maintenance in culture (**C**) changes the appearance of the chronic cells from flattened and spread out to more bi-polar (and thereby more like the appearance of the NF cells). Scale bar = 20 µm.

To confirm that telomerase was ectopically expressed in the CWF-hTERT and NF-hTERT that underwent lifespan extension, a number of analyses were undertaken. RT-PCR demonstrated that both the constitutive template component (hTR) and catalytic component (hTERT) were expressed in both of the cell types (Figure 2A). The telomere repeat amplification protocol (TRAP) assay was performed on genomic DNA isolated from cell populations. Consistent with the observations of extended lifespan, all the populations of immortalized cells demonstrated ectopic telomerase activity, as demonstrated by a discernible 6 bp laddering, corresponding to multiples of the TTAGGG repeats of the telomere sequence (Figure 2B). Such telomerase activity was coupled with a distinct alteration in the appearance of the CWF (Figure 2C). Primary CWF strains, even at low passage/population doubling level, were enlarged and stellate in appearance; features that were not attributable to the equivalent passage/patient-matched NF, which were smaller and bi-polar in appearance. However, post immortalisation and with increasing time in culture, the morphology of the CWF-hTERT cells changed to become more like the morphology of NF-hTERT cells (i.e., they transitioned from a chronic appearance to a more ‘normal’ appearance).

### 2.3. The Immortalisation Process Does Not Distort Gene Expression Signatures

As the aim of this investigation was to validate the establishment of a disease-specific, immortalised cell resource, a global gene expression analysis was undertaken to determine whether immortalisation using hTERT alters gene expression (i.e., the gene expression profile of the immortalised cell lines is similar to the primary cell strains). Affymetrix microarray analysis was undertaken on both primary CWF and NF and on hTERT immortalised CWF and NF at 0 h (serum starved) and 6 h (post serum stimulation), and at three culture time points after immortalisation (P23 [an early culture time point post immortalisation], P50 [a mid-culture time point post immortalisation), and P80 [a late culture time point post immortalisation]).

Primary fibroblasts were compared to hTERT immortalised fibroblasts irrespective of source, time post-immortalisation, or serum stimulation status. Using a false discovery rate (FDR) of 0.05 only six significantly altered genes were identified as being altered by immortalisation (Table 2). When the FDR was relaxed to 0.01 a number of additional genes were identified as being altered by immortalisation (Table 3). When over-representation analysis was carried out using these gene lists the only pathways/processes significantly over represented in the genes altered by immortalisation were associated with cell proliferation (Cell Cycle, Mitosis, and DNA Replication) or telomere maintenance (Table 4). As hTERT immortalised cells are capable of sustained proliferation (exemplified in Figure 1) the identification of genes involved in cell cycle, DNA replication, and telomere maintenance as being altered by hTERT immortalisation was expected. Overall, therefore, whilst there were some limited changes in the expression of individual genes between the primary and immortalized cells, there were no major changes to the transcriptional profile of the cell lines when viewed from the process level (i.e., the immortalization process did not have any effect on processes/pathways that would not have been expected to be altered).

### 2.4. Immortalisation Does Not Reverse the Disease-Specific Wound Healing Cellular Phenotype

**Figure 3:**
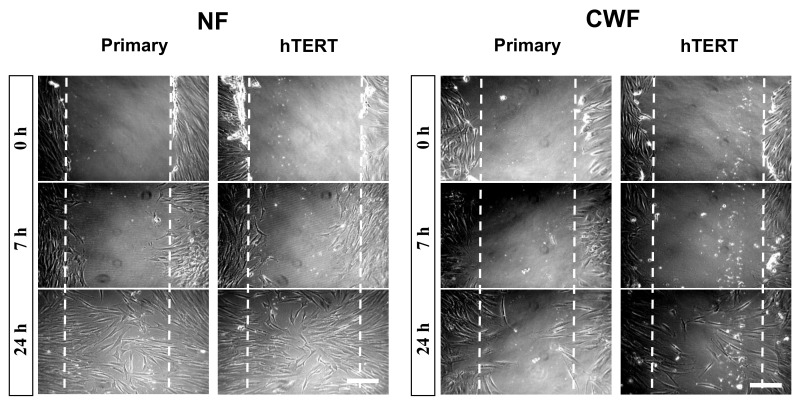
Utilising a monolayer wound scratch assay and time lapse microscopic analysis immortalised NF, like the primary cells from which they were established, clearly repopulated the wound space over a period of 24 h whereas the patient-matched CWF cells (both primary and immortalised) failed to do so. Scale bar = 100 µm.

Fundamental to the development of any immortalised, disease-specific cell line is that the immortalisation process does not reverse the disease-related cellular phenotype. As demonstrated in Table 1, there were maintained CWF and NF differences in proliferative rate; cell proliferation being a key phenotypic response during wound healing. Another key wound repair property is wound repopulation therefore the ability of the cell populations to undertake this crucial response was assessed using a monolayer scratch wound assay and time-lapse microscopy. As reported previously for the primary cell strains [22], there was a distinct difference in wound repopulation ability; NF repopulated the wound after 24 h, but the CWF failed to do so (Figure 3; Supplemental Movies 1 & 2). Importantly, such differences in wound repopulation were also maintained with their immortalised counterparts (Figure 3; Supplemental Movies 3 & 4).

## 3. Discussion

This investigation has described the characterisation of hTERT immortalised disease and normal fibroblast cell lines. These cell lines were derived from primary cells strains isolated from both chronic wounds and patient matched normal skin. The aetiology of venous leg ulcers is poorly understood and as such it is difficult to generate accurate model systems to permit the screening of candidate therapeutic interventions. In an attempt to replicate the chronic wound environment, animal models have been established, but these are really models of impaired, rather than chronic, wound healing. Whilst primary human cell strains taken from chronic wounds have been utilised for investigations in the past [22,23,24,25,26,27,28,29] these cells have a limited life span and reproducibility in the laboratory, and therefore continual patient samples are necessitated. Furthermore, due to the variation between cell isolations from different patients, it can be difficult to obtain reliable and reproducible data when using multiple primary isolations. Therefore, the aim of this study was to characterise immortalised CWF and NF cell lines to provide a robust research tool that will be suitable for (a) investigating the biology of chronic wounds and (b) the future screening of candidate therapeutics for chronic wounds.

This study has confirmed that the delivery of hTERT enabled the cells to escape replicative senescence, as demonstrated by their extended lifespan. Non-immortalised CWF could achieve a maximum of around 40 PD, yet hTERT-immortalised CWFs were still growing past 80 PD. TRAP assay and PCR analysis confirmed such telomerase activity. Crucially, we also immortalised patient-matched NF in order to account for inter-patient variability; these cells were also telomerase positive and were still growing at 120 PD (more than twice the reported Hayflick limit [38]). Importantly, in line with the observed differences in the CWF and NF cell strains prior to immortalisation, overall the hTERT immortalised CWFs demonstrated reduced population doubling rates when compared to their patient matched hTERT NFs. This suggests that even though the senescence barrier has been overcome, there are still distinct phenotypic anomalies that are intrinsic to the diseased cell lines.

To determine if immortalisation significantly altered gene expression, microarrays were used to compare the hTERT immortalised cells with the primary cells from which they were derived at three time points after immortalisation. The number of genes altered by immortalisation was small and few known pathways were affected. Within the CWF and NF, over representation analysis of these genes altered by the immortalisation process unsurprisingly identified pathways that were relating to the cell cycle and DNA replication. These findings correlate with the increased proliferative ability of the immortalised cells. Such an alteration in a limited number of genes (often in association with the cell cycle) is supported by the findings of others [46] and agrees with reports that demonstrate a good correlation between immortalised and parental cells [47]. Hence, these stable gene expression signatures validate the hTERT immortalised cell lines as being an appropriate model of the primary cell strains from which they were generated.

However, not all studies of hTERT immortalised fibroblast cell lines agree as to the effect hTERT has on cellular phenotype. Lindvall et al. described significant differences in gene expression in their hTERT-immortalised cells, including DNA repair genes and growth factor genes [48]. Wang et al. (2000) showed hTERT immortalisation activated the oncogene c-MYC [49] and other oncognenes including p16 and p53 have also been reported to be upregulated [50,51], something that would not be desired if the cells were to be used for direct therapeutic interventions. Other studies, however, contradict these findings. Pirzio et al. (2004) demonstrated low frequencies of chromosomal aberrations in hTERT immortalised fibroblasts suggesting a role in genome stability for telomerase [52]. Furthermore, Milyavsky et al. reported an up-regulation of p16 in their hTERT immortalised cells and the retention of chromosome stability and the ability to enter G0 [53]. Importantly, none of these gene expression alterations described previously were observed in any of the patient matched NF or CWF cell lines investigated in this study.

Importantly, after immortalisation CWF retained their reduced ability to repopulate scratch wounds. This reduced wound repopulation capacity is a characteristic previously reported for primary CWF [22]. Wound repopulation in the monolayer scratch wound assay is via a combination of proliferation and migration. In this study, the hTERT-CWF had a somewhat reduced growth rate when compared to that of hTERT-NF so it is likely that the reduced repopulation is due to impaired proliferation and migration (a specific disease phenotype still maintained after immortalisation). This is significant in the context of wound healing because cells that have an impaired ability to repopulate a wound environment in vivo will have a reduced capacity to adequately repair that tissue.

## 4. Materials and Methods

### 4.1. Patients and Tissues

Cultures of CWFs and patient-matched, uninvolved NFs were established following approval from the Local Research Ethics Committee and after written informed consent from patients (*n* = 4) with non-healing, chronic venous leg ulcers attending the Wound Healing Clinic at the University Hospital of Wales, Cardiff. Only patients with wounds that failed to respond to conventional treatment regimes after two months were used in the study; patients with diabetes, systemic immunosuppression, or clinical signs of local infection were excluded. A 6-mm biopsy was taken from the chronic wound bed and the uninvolved outer aspect of the ipsilateral thigh. All of the experiments were carried out according to the Declaration of Helsinki Principles.

### 4.2. Establishment of Immortalized Chronic Wound and Patient-Matched NFs

hTERT immortalised fibroblast cell lines were generated from chronic wound and patient matched normal fibroblast cell strains (strains described previously [22]). Fibroblasts were transfected with the hTERT containing retroviral vector pBABE-hTERT. Positively transfected cells were selected by the addition of puromycin to the growth medium (Fibroblast-Serum Containing Medium [F-SCM + Puro], consisting of Dulbecco’s Modified Eagle’s Medium (DMEM) supplemented with l-glutamine (2 mM), antibiotics (100 U/mL penicillin G; 100 µg/mL streptomycin sulphate; 0.25 µg/mL amphotericin B), puromycin 1 µg/mL, and 10% (*v*/*v*) foetal calf serum [FCS]; all of the reagents supplied by Invitrogen, Paisley, UK). Upon reaching 80–90% confluence, fibroblast populations were passaged at a ratio of 1:3.

The populations doublings (PDs) of the cell populations were derived by direct counting of cell numbers at each passage then calculation using the method previously described [54]. Cells in monolayer culture were digitally imaged 24 h after passage using a Nikon Coolpix 995 digital camera (Nikon, Kingston-upon-Thames, UK) attached to an Olympus CK2 microscope (×10 objective lens, F-Stop 3.5) (Olympus, Southall, UK).

### 4.3. Telomerase Repeat Amplification Protocol (TRAP) Assay

Protein extracts were prepared and the TRAP assay was performed as described previously [55] with some modifications. Cells (5 × 10^5^) were re-suspended in 1 mL wash buffer (10 mM Hepes-KOH, 1.5 mM MgCl_2_, 1 mM KCl, and 1 mM DTT) and spun at 15,000× *g* for 2 min 4 °C. The supernatant was removed and the cells were re-suspended in 100 µL lysis buffer (10 mM Tris-HCl, 1.5 mM MgCl_2_, 1 mM EGTA, 10% glycerol, 0.5% CHAPS, 1 mM PMSF, and 0.35% 2-mercaptoethanol). Cells were incubated on ice for 30 min. The lysate was then centrifuged at 20,000× *g* for 30 min at 4 °C and the supernatant collected and frozen on dry ice in 10 µL aliquots.

Reactions were set up in RNase free 0.5 mL microtubes, each reaction containing 2 µL of protein extract and 48 µL of 1× reaction mix (40 mM Tris-HCl, 3 mM MgCl_2_, 126 mM KCl, 0.01% Tween 20, 2 mM EGTA, 0.2 g/L BSA, 100 M dNTPs, 1 µg T4 gene 32 protein and 100 ng TS primer). Negative controls for each reaction were set up with heat denatured protein extracts (10 min at 85 °C). Reactions were incubated for 30 min at 30 °C, the temperature was increased to 92 °C and 100 ng CX primer, and 2.5 U Taq polymerase were added to each reaction. TRAP products were amplified by 31 cycles (92 °C for 30 s, 50 °C for 30 s, and 72 °C for 90 s). TRAP products were run on a 10% polyacrylamide (19:1) and visualised using Sybr Gold (Invitrogen) and a Typhoon 9400 Variable Mode Imager (GE Healthcare, Little Chalfont, UK) using an excitation wavelength of 488 nm and a 520 BP40 emission filter.

### 4.4. Reverse Transcription Polymerase Chain Reaction

PCR reactions were set up with the resulting cDNA and using the following primers: TR: 5-CTA ACC CTA ACT GAG AAG GGC GTA-3 (TRC3F) and 5-GGC GAA CGG GCC AGC AGC TGA CAT T-3 (TRC3R [56]) TERT: 5-CGG AAG AGT GTC TGG AGC AA-39 (LT5) and 5-GGA TGA AGC GGA GTC TGG A-3 (LT6 [57]). As a control for RNA quality and successful cDNA synthesis, the GAPDH gene was amplified using specific primers, including 5-CTC AGA CAC CAT GGG GAA GGT GA-39 (K136) and 5-ATG ATC TTG AGG CTG TTG TCA TA-39 (K137). The PCR conditions used for the amplification of these genes were: initial incubation at 94 °C for 10 min, 36 cycles with 94 °C for 20 s, step down annealing from 60 to 55 °C for 20 s, 72 °C for 20 s, and a final incubation at 72 °C for 10 min. PCR products were run on a 2% agarose gel and visualized by ethidium bromide staining.

### 4.5. Microarray Analysis

RNA was extracted from serum-induced cells from all four patients. Briefly, cells were seeded at a density of 640 cells cm^2^ in 20 cm diameter TC dishes and were cultured under standard conditions for 24 h. Cells were then serum-starved for 48 h to induce quiescence. Cells were then re-stimulated with serum and harvested at 0 and 6 h for RNA extraction. Cells were washed with phosphate-buffered saline, incubated with TRIzol (Invitrogen) at room temperature for 10 min, and RNA was extracted according to standard phenol/chloroform extraction protocols. RNA concentration was quantified on an automated RNA quantitation machine (Bio-Rad) and RNA quality was determined using the RNA Nano LabChip kit (Agilent Technologies, Wokingham, UK). Samples were then processed by the Central Biotechnology Service (Cardiff University, Cardiff, UK), following the detailed protocol for sample preparation and microarray processing available from Affymetrix (http://www.affymetrix.com). Briefly, first-strand cDNA was synthesized from 5 µg total RNA using a T7-(dT)24 primer (Genset Corp., Nottingham, UK) and reverse-transcribed with the Superscript Double-Stranded cDNA Synthesis Kit (Invitrogen). After second-strand synthesis, the resulting cDNA was subjected to an in vitro transcription reaction using a Bioarray kit (Enzo Diagnostics, New York, NY, USA) to generate biotinylated cRNA. This was subsequently fragmented and hybridized to the Affymetrix U133A GeneChip, which contains approximately 22,000 probe sets to detect the transcripts of approximately 14,000 distinct human genes, plus additional expressed sequences tags.

Following hybridization and scanning, the resultant image files (.CEL) were analyzed using the MAS 5.0 statistical algorithm (Affymetrix) to calculate an expression summary value for each probe set on each GeneChip. The resulting expression data were scaled to a target intensity of 100. Differentially expressed genes were identified by applying analysis of variance to the expression values for each probe set, using (i) the patient identifier, (ii) the cell type (CWF vs. NF), and (iii) ±serum treatment as the three sets of factors. The resulting *p*-values were corrected for multiple hypothesis testing using the false discovery rate method [58]. The expression values were further visualized and explored by hierarchical clustering (cosine similarity applied to log-transformed, median-centred data) and heat map visualization.

*Difference of the differences* testing was undertaken using expression summaries that were generated using the RMA algorithm [59]. For each patient NF or CWF sample, the (log2) fold change between each pair of samples with and without serum was first calculated and then mean values for the NF and CWF groups were calculated. Probe sets were selected where the mean induction (or repression) by serum was twofold greater in the CWF group when compared with the NF group. All of the analyses were performed in R and the scripts for all of the analyses are available from the authors upon request. Over representation analysis was undertaken using the GOstats packages in R, using gene sets derived from the Gene Ontology Biological Processes dataset.

### 4.6. In Vitro Wounding Studies

A standard monolayer scratch wound model was used to characterize wound repopulation. Briefly, cells were seeded into 24-well tissue culture plates (2 × 10^4^ cells per well), cultured to confluence, and monolayers were wounded by scratching along the surface of the tissue culture plastic with a standard 200 µL pipette tip. Cellular monolayers were gently washed with phosphate-buffered saline, re-fed with F-SCM, and incubated under standard culture conditions on the motorized, heated, and gassed stage of a Zeiss Axiovert 200 M microscope (Carl Zeiss, Welwyn Garden City, UK). Images were collected every 15 min for 40 h using the Openlab software package, which was also used for post-image acquisition analysis (Improvision, Coventry, UK).

## 5. Conclusions

hTERT-immortalised CWF (and patient-matched NF) cell lines have been established and characterised with respect to their phenotype and genotype. The immortalisation process was demonstrated to be effective as cells expressed telomerase and had extended lifespans. Furthermore, immortalisation of NF and CWF retained genotypic and phenotypic differences. Validated disease cell lines are now available for use in future studies of chronic wound healing. It is envisaged that these cell lines may eventually offer a real alternative to the use of animal models in chronic wound research and be a useful research tool for understanding the aetiology of chronic wounds as well as a potential high-throughput screening tool for candidate therapeutics that can ameliorate the chronic wound phenotype.

## Figures and Tables

**Table 1:** Respective growth rates of the CWF and NF cell lines and strain.

**Days**	**NF Primary Patient 1**	**CWF Primary Patient 1**	**NF Mock Patient 1**	**CWF Mock Patient 1**	**NF hTERT Patient 1**	**CWF hTERT Patient 1**
0–50	2.660395186	1.372377017	2.903460321	1.676095021	2.820859002	1.761228228
50–100	2.063483514	1.155055922	2.21362413	1.491181981	2.17190845	2.350465441
100–150	1.708425038	0.238615271	1.931265023	0.808847164	2.888996473	2.100595582
150–200	1.092771348	-	1.209523855	0.589792188	2.546683912	2.147975214
200–250	0.65051411	-	0.306199448	-	2.911358566	2.340073042
250–300	-	-	-	-	3.386106828	1.914658417
300–350	-	-	-	-	4.037277255	2.316985136
**Days**	**NF Primary Patient 2**	**CWF Primary Patient 2**	**NF Mock Patient 2**	**CWF Mock Patient 2**	**NF hTERT Patient 2**	**CWF hTERT Patient 2**
0–50	2.00003978	1.430818888	2.702882371	1.408288338	2.702421121	1.315226122
50–100	1.897508302	0.642582236	2.506688459	0.389073493	2.084423303	1.526944077
100–150	2.048256328	-	1.937150809	-	3.091048229	2.73604129
150–200	1.362063393	-	0.741986217	-	2.848323718	2.544191407
200–250	0.468038965	-	-	-	2.975591664	3.613521307
250–300	0.225106176	-	-	-	3.282693337	3.238706858
300–350	-	-	-	-	3.283963285	3.136433714
**Days**	**NF Primary Patient 3**	**CWF Primary Patient 3**	**NF Mock Patient 3**	**CWF Mock Patient 3**	**NF hTERT Patient 3**	**CWF hTERT Patient 3**
0–50	1.773899657	1.838731796	1.876174641	1.811924471	1.864799274	1.957757805
50–100	2.137331544	1.820469606	2.227068604	1.765460977	2.175212065	1.931044873
100–150	1.700143374	1.313762689	1.821538813	1.463326161	2.040014915	1.948431103
150–200	1.359040254	1.018979963	1.30906374	0.656850067	1.866400168	1.991061535
200–250	0.96345786	0.381403418	0.386622971	0.60575738	3.009158197	1.847844344
250–300	0.494600255	-	-	-	2.453037313	1.455326622
300–350	0.918257283	-	-	-	2.36463098	1.521344782

**Table 2:** Counts of genes identified as significantly different (FDR 0.05).

	Normal (NF)				Wound (CWF)			
	*0 h*		*6 h after Serum Stimulation*		*0 h*		*6 h after Serum Stimulation*	
**Early**	FDR 0.05	0	FDR 0.05	0	FDR 0.05	0	FDR 0.05	0
**Mid**	FDR 0.05	0	FDR 0.05	6	FDR 0.05	0	FDR 0.05	0
**Late**	FDR 0.05	3	FDR 0.05	0	FDR 0.05	0	FDR 0.05	0

**Table 3:** Counts of genes identified as significantly different (FDR 0.01).

	Normal (NF)				Wound (CWF)			
	*0 h*		*6 h after Serum Stimulation*		*0 h*		*6 h after Serum Stimulation*	
**Early**	FDR 0.01	476	FDR 0.01	193	FDR 0.01	161	FDR 0.01	145
**Mid**	FDR 0.01	653	FDR 0.01	866	FDR 0.01	292	FDR 0.01	203
**Late**	FDR 0.01	418	FDR 0.01	507	FDR 0.01	446	FDR 0.01	660

**Table 4:** Pathways/processes that were significantly over represented after immortalisation, that is were up-regulated in hTERT cells (i.e., only those associated with cell proliferation [Cell Cycle, Mitosis and DNA Replication] or telomere maintenance were altered).

Pathway	Genes
DNA replication	TOP2A, MCM3, RRM2, MCM6, NASP, CDC6, RFC3, CDK2, FEN1, CHEK1, POLE2, GINS1, TERT, MCM7, NF2, CIZ1, GMNN, DTL, PDGFC, MCM4
DNA strand elongation involved in DNA replication	MCM3, MCM6, RFC3, FEN1, GINS1, MCM7, MCM4
cell cycle	TOP2A, MCM3, RANBP2, RRM2, MCM6, NASP, TUBB3, GAS7, CCNA2, TFDP2, FGFR2, RARA, MAPRE3, CDC6, ZWINT, RFC3, CKS2, CDK2, RASSF1, FEN1, BARD1, CHEK1, POLE2, GINS1, TUBB2C, HMG20B, SMAD6, TGFB2, TPX2, MCM7, KIF2C, TUBB, CDKN2C, ARAP1, CDKN1C, CKAP2, GMNN, CEP55, DTL, MCM4, RACGAP1
cell cycle phase	POLD2, TOP2A, PSMD3, MCM3, RRM2, CHMP1A, TUBB3, DNM2, RANBP1, UBE2S, RFC5, CDK1, CAV2, MAD2L1, CCNA2, BUB1B, DLGAP5, CDC6, ZWINT, TRIP13, AURKA, CKS2, DBF4, CDK2, PSMB9, GTSE1, CDC7, KIF23, FEN1, CCNF, CENPA, GINS1, AKT1, DGKZ, TUBB2C, TUBB, ID4, BUB1, CDKN3, HGF, TPX2, CDKN2D, SSNA1, MCM7, KIF2C, KPNA2, RANGAP1, TFDP1, NEK1, TCF3, MDM2, PRC1, NUSAP1, GMNN, CEP55, PBK, CDKN1C, CENPN, NCAPG2, TBRG4, CCDC99, MCM4
organelle fission	CHMP1A, TUBB3, RANBP1, CDK1, CAV2, MAD2L1, CCNA2, BUB1B, DLGAP5, CDC6, ZWINT, AURKA, CDK2, KIF23, CCNF, CENPA, TUBB, BUB1, HGF, TPX2, KIF2C, RANGAP1, NEK1, NUSAP1, CEP55, PBK, CENPN, NCAPG2, CCDC99
mitotic cell cycle	POLD2, TOP2A, PSMD3, MCM3, RRM2, CHMP1A, TUBB3, DNM2, RANBP1, UBE2S, RFC5, CDK1, CAV2, MAD2L1, CCNA2, BUB1B, DLGAP5, CDC6, ZWINT, AURKA, DBF4, CDK2, PSMB9, GTSE1, CDC7, KIF23, FEN1, CCNF, CENPA, GINS1, AKT1, DGKZ, TUBB2C, TUBB, ID4, BUB1, CDKN3, HGF, TPX2, CDKN2D, SSNA1, MCM7, KIF2C, KPNA2, RANGAP1, TFDP1, NEK1, TCF3, MDM2, PRC1, NUSAP1, GMNN, CEP55, PBK, CDKN1C, CENPN, NCAPG2, TBRG4, CCDC99, MCM4
cell division	PPP1CA, CHMP1A, UBE2S, CDK1, MAD2L1, CCNA2, FGFR2, BUB1B, CDC6, ZWINT, AURKA, CKS2, CDK2, CDC7, KIF23, CCNF, FGF1, FGF5, MDK, PTN, BUB1, TPX2, KIF2C, NEK1, PRC1, NUSAP1, CEP55, PDGFC, PDGFD, NCAPG2, CCDC99, RACGAP1
mitosis	CHMP1A, TUBB3, RANBP1, CDK1, CAV2, MAD2L1, CCNA2, BUB1B, DLGAP5, CDC6, ZWINT, AURKA, CDK2, KIF23, CCNF, CENPA, TUBB, BUB1, HGF, TPX2, KIF2C, RANGAP1, NEK1, NUSAP1, CEP55, PBK, CENPN, NCAPG2, CCDC99
M phase	TOP2A, CHMP1A, TUBB3, RANBP1, UBE2S, CDK1, CAV2, MAD2L1, CCNA2, BUB1B, DLGAP5, CDC6, ZWINT, TRIP13, AURKA, CKS2, CDK2, KIF23, CCNF, CENPA, TUBB, BUB1, HGF, TPX2, KIF2C, KPNA2, RANGAP1, NEK1, PRC1, NUSAP1, CEP55, PBK, CENPN, NCAPG2, CCDC99
DNA replication	RFC2, POLD2, PCNA, TOP2A, RRM1, RPA1, MCM3, MCM5, RRM2, MCM6, NASP, MCM2, TK1, TYMS, RNASEH2A, RFC5, CDC6, RFC4, RFC3, DBF4, POLA2, FEN1, CHAF1B, POLA1, RAD51, CCNE2, CHEK1, PDGFA, CIZ1, GINS1, TERT, CENPF, MCM7, DUT, CDT1, NF2, ORC5, MCM4, CDC34, GMNN, DTL, RMI1, TIPIN, MCM10, GINS2
cell cycle checkpoint	RFC2, TOP2A, BUB3, RPA1, MCM3, MCM5, MCM6, BIRC5, MCM2, CCNB2, CDC20, UBE2C, RFC5, CDK1, MAD2L1, CCNA2, FANCG, BUB1B, CDC6, RFC4, ZWINT, RFC3, DBF4, PSMB9, GTSE1, TTK, POLA1, CCNE2, CHEK1, CENPF, MCM7, BUB1, CDT1, ORC5, MCM4, CCNB1, ZWILCH, DTL, TIPIN, MCM10
cell division	PPP1CA, BUB3, NCAPD2, CDC25B, PSRC1, BIRC5, CCNB2, CDC20, UBE2C, CDK1, MAD2L1, CCNA2, FGFR2, BUB1B, CDC6, ZWINT, AURKA, NDC80, CKS2, SMC2, KIF11, NEK2, KIF23, CCNF, CCNE2, RAB35, PDGFA, DIAPH2, CENPF, MAEA, FGF5, MDK, KIF2C, AURKB, PTN, BUB1, TGFB2, TPX2, OIP5, CCNB1, PRC1, NUSAP1, ZWILCH, CEP55, FBXO5, TIPIN, NCAPG2, ERCC6L, ASPM, CDCA3, CDCA8, RACGAP1
S phase of mitotic cell cycle	RFC2, POLD2, PCNA, RPA1, MCM3, MCM5, MCM6, MCM2, BCL6, RFC5, CDC6, RFC4, RFC3, PSMB9, POLA2, FEN1, POLA1, GINS1, MCM7, CDT1, ORC5, MCM4, GINS2
telomere maintenance via recombination	RFC2, POLD2, PCNA, RPA1, RFC5, RFC4, RFC3, POLA2, FEN1, POLA1
telomere maintenance via semi-conservative replication	RFC2, POLD2, PCNA, RPA1, RFC5, RFC4, RFC3, POLA2, FEN1, POLA1
M/G1 transition of mitotic cell cycle	RPA1, MCM3, MCM5, MCM6, MCM2, CDC6, DBF4, PSMB9, POLA2, POLA1, MCM7, CDT1, ORC5, MCM4, GMNN, MCM10

## Supplementary Materials

Supplementary materials can be found at http://www.mdpi.com/1422-0067/19/4/1001/s1. **Sup Video 1 NF:** Scratch wound repopulation by NF cell strain; **Sup Video 2 CWF:** Scratch wound repopulation by CWF cell strain; **Sup Video 3 hTERT NF:** Scratch wound repopulation by NF cell line; **Sup Video 4 hTERT CWF:** Scratch wound repopulation by CWF cell line.
